# The effect of exergame rehabilitation on the quality of life of cancer patients undergoing abdominal surgery: a randomized controlled trial

**DOI:** 10.1007/s00520-024-09005-0

**Published:** 2024-11-15

**Authors:** Isabel Alves, Ana Paula Moreira, Teresa Sousa, Paulo Teles, Bruno Miguel Magalhães, Filipe Goncalves, Carla Silvia Fernandes

**Affiliations:** 1 Portuguese Institute of Oncology, Porto, Portugal; 2 School of Economics, University of Porto, Dr. Roberto Frias, 4200-464 Porto, Portugal; 3 Porto Higher Health School of Health, University of Trás-Os-Montes E Alto Douro (UTAD), Vila Real, Portugal; 4 Comprehensive Cancer Centre (Porto.CCC) RISE@CI-IPOP (Health Research Network), Porto, Portugal; 5 Faculty of Health Sciences, University of A Coruña, A Coruña, Spain; 6 APELA - Portuguese Amyotrophic Lateral Sclerosis Association, Porto, Portugal; 7 Porto Higher School of Nursing, Porto, Portugal; 8 ADITGames Association, Porto, Portugal

**Keywords:** Games, Quality of life, Exergames, Neoplasms, Rehabilitation

## Abstract

**Purpose:**

Exergames, which combine digital games and physical exercise, have become increasingly popular for rehabilitation in the health domain. This study aimed to assess the effectiveness of exergame rehabilitation on the quality of life of cancer patients undergoing abdominal surgery.

**Methods:**

This randomized controlled trial evaluated the effectiveness of exergame rehabilitation on the quality of life of cancer patients who had undergone abdominal surgery. Seventy postoperative patients were included, and data collection took place between January 2023 and May 2023. The patients were randomly assigned to either an exergame rehabilitation program (*n* = 35) or a traditional rehabilitation program (*n* = 35). The assessed outcome was the quality of life, and data collection occurred at three different time points: upon admission, 48 h postoperatively, and on the 7th day after surgery.

**Results:**

Quality of life was evaluated using the WHOQOL-BREF Scale. At the third assessment, a statistically significant difference was observed between the two groups (*p* = 0.016), indicating that the intervention group had a higher quality of life than the control group.

**Conclusions:**

The study showed a positive effect of exergames on the population under investigation. By the 7th day after surgery, the intervention group demonstrated an improvement in their quality of life compared to the control group.

**Clinical trial registration:**

Center of Open Science OSF https://osf.io/286zb/, registered in July, 2023.

## Introduction

Surgery is considered the primary treatment for cancer in numerous situations [1]. Patients undergoing abdominal cancer surgery for gastrointestinal, urological, gynecological, hepatobiliary, and pancreatic malignancies constitute a group that requires special attention, especially as factors such as cachexia, myopenia, and sarcopenia have been shown to be associated with worse medium- and long-term outcomes [2]. In the case of abdominal surgery, pain is one of the disruptive symptoms causing considerable discomfort, which may hinder the rehabilitation process [3]. Additionally, surgery or general anesthesia can potentially cause visceral organ paralysis, making the main postoperative care objectives to help individuals manage postoperative symptoms, promote mobilization and physical rehabilitation, and restore function to the highest possible level [1, 4]. All these factors can negatively impact patients’ quality of life.

Oncological rehabilitation aims to improve the quality of life of cancer patients, helping them adapt to a lifestyle as close as possible to what they were accustomed to before the disease. Therefore, early mobilization in the postoperative period is highly recommended [5]. Apart from the inherent issues of any surgery, cancer treatment can generate adverse effects, and each type of treatment can lead to different sequelae, such as nausea, changes in major organs, decreased bone density, reduced muscle strength, impaired physical fitness, and alterations in cardiac and pulmonary function [4, 6]. People with cancer experience reduced quality of life, functionality, range of motion, and strength, and an increase in pain and fatigue [3, 7]. Patients’ expectations regarding surgical recovery are related not only to the treatment itself but also to a strong emphasis on measures of the person’s quality of life, where traditional surgical and oncological outcomes are complemented with measures of satisfaction and overall health-related quality of life (HRQL) [8].

In this context, results can be enhanced by minimizing complications through an early rehabilitation program focused on physical mobilization [1]. Therefore, engaging in physical activities can improve physical and emotional performance, consequently enhancing the quality of life of cancer patients during and after treatments such as chemotherapy, radiotherapy, and surgery [4, 6].

In recent years, there has been a trend toward the use of games in various health-related situations. Interactive video games, also known as exergames, allow the individual to interact with the game by moving limbs or the entire body [6]. Also known as exergaming, defined as the combination of exercise and gaming, it is a relatively new idea in rehabilitation where the user must use physical movements to interact with a game [7]. These resources have also been considered a fun and enjoyable method of physical activity, potentially increasing motivation to participate in exercise and rehabilitation programs [9]. The benefits of exergames in other clinical populations have been increasingly recognized [10–13], but there is limited research evaluating the feasibility, acceptability, and efficacy of exergame interventions among patients with current or previous cancer diagnoses [7, 14] and absent specifically in oncological abdominal surgery rehabilitation. Moreover, the potential of these technologies could help motivate and challenge individuals. Rehabilitation is crucial after surgery for cancer patients, and the utilization of exergames could serve as a significant resource for postoperative recovery [14]. Incorporating exergames into rehabilitation enables the promotion of greater intensity in rehabilitation exercises without the sensation of excessive effort [9]. However, it is important to highlight the conclusions of the review study conducted on this topic, where the authors stated that no negative impacts were reported in any of the various studies [14]. Understanding the potential of these resources, we have undertaken a randomized study with a control group to assess the effectiveness of exergaming on the quality of life of patients after abdominal oncological surgery.

## Methods

### Study design

**Fig. 1:**
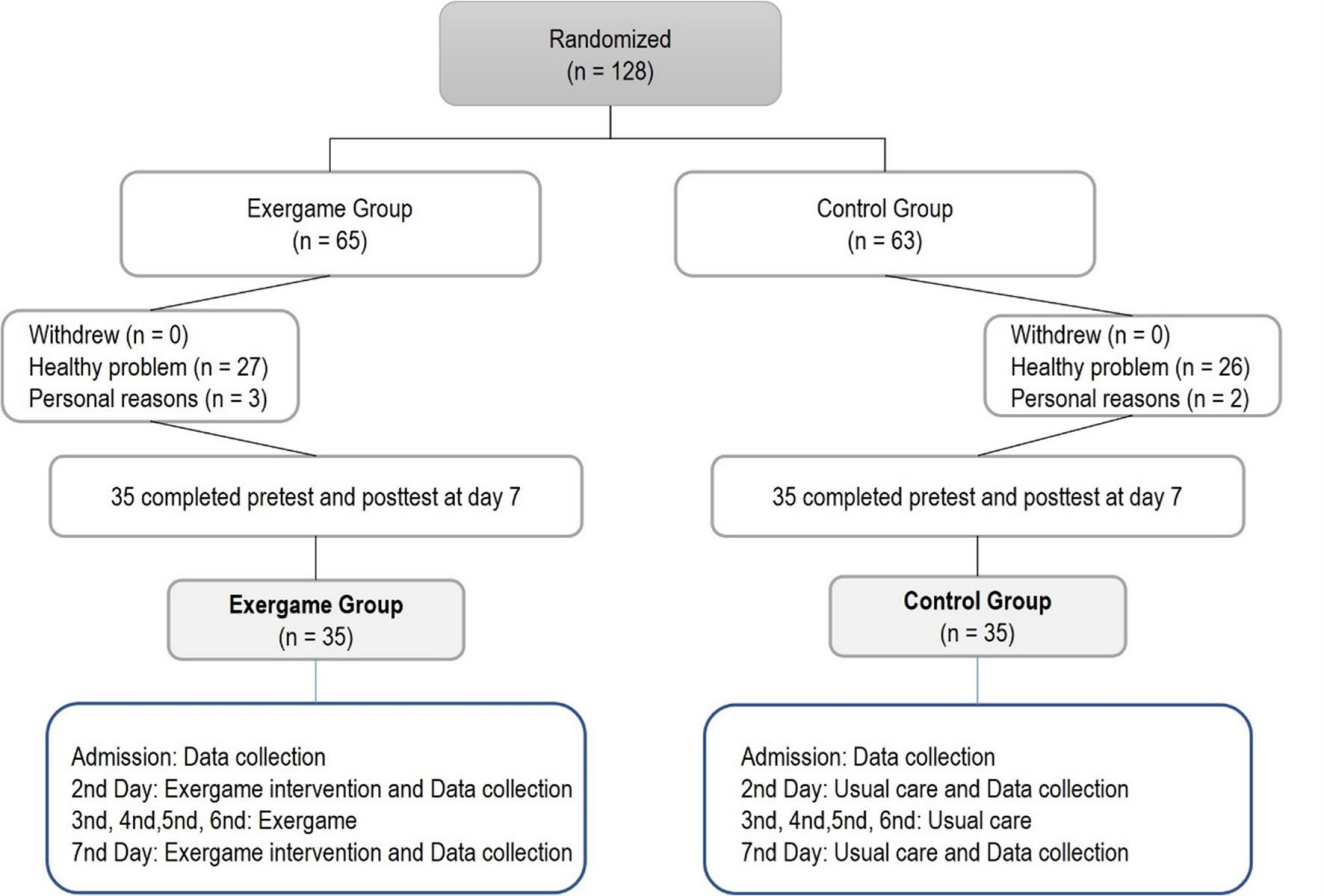
Study design

The randomized controlled trial evaluated the impact of exergame rehabilitation on the quality of life of oncology patients undergoing surgery, using pre- and post-tests. The study was conducted with two arms, and all patients were recruited from two Surgery departments at an Oncology Hospital in Portugal. Standard postoperative treatment was applied in the control group, while the intervention group received the intervention using exergames (Fig. 1). Randomization took place upon patient admission, with control group patients scheduled for surgery on even-numbered days and intervention group patients on odd-numbered days. Both groups were treated identically in all aspects except for the intervention. This study was conducted following the CONSORT guidelines [15] (https://osf.io/286zb/).

### Participants

Seventy-five patients undergoing major abdominal surgery were recruited between January 2023 and May 2023. Five patients refused to take part in the study. Due to the limited number of robust previous studies for this patient population, it was not possible to calculate the sample size based on known data [12]. Therefore, the sample size was calculated using an estimated effect size, considering 0.80 as the minimum “clinically” relevant difference between the groups [16]. The sample size was calculated using an analysis of the mean difference between two groups using the G*Power® program. A significance level of 5% and a test power of 80% were adopted. The choice of this effect size informed our sample size calculation, ensuring that we have an adequate number of participants to detect a significant difference with sufficient statistical power.

Thirty-five patients participated in the control group, and 35 were in the intervention group. The following inclusion criteria were applied: patients with scheduled abdominal surgery and expected hospitalization the day before surgery; conscious and oriented patients in terms of time and space; patients who agreed to participate in the study with informed consent.

As for the exclusion criteria, the following aspects were considered: patients with cognitive deficits; patients with motor deficits (e.g., hemiparesis); patients with severe visual impairment (not correctable with glasses/lenses); patients with clinical indications for not performing lifting or mobilization; patients with a request for admission to the intensive care unit (ICU) or intermediate care unit (IMCU); patients with scheduled abdominal surgery for diagnostic/staging purposes (exploratory laparotomies/staging laparoscopies) (Table 1.).

**Table 1:** Participants characteristics

Variables		Control (*n*=35)			Intervention (*n*=35)			*P*-value
		*n*	%	Mean	*n*	%	Mean	
Age				62.3			63.1	0.823
BMI				25.21			25.28	0.66
Hospitalization Length (Days)				8.4			7.7	0.621
Gender	Male	25	71.4		20	57.1		0.318
	Female	10	28.6		15	42.9		
Marital Status	Single	0	0.0		6	17.1		0.001
	Married/Union	31	88.6		27	77.1		
	Divorced	2	5.7		1	2.9		
	Widowed	2	5.7		1	2.9		
Admission Diagnosis	Intestinal Cancer	25	71.4		20	57.1		0.78
	Gastric Cancer	7	20.0		12	34.3		
	Other	3	8.6		3	8.6		
Surgery	Colectomy	14	40.0		13	37.1		0.77
	Gastrectomy	5	14.3		11	31.4		
	Abdomino-perineal amputation	4	11.4		3	8.6		
	Hepatic Mestastasectomy	3	8.6		0	0		
	Other	9	25.7		8	22.9		
Metastatic Disease	Yes	11	31.4		6	17.1		0.265
	No	24	68.6		29	82.9		
Chemotherapy	Yes	10	28.6		11	31.4		0.999
	No	25	71.4		24	68.6		
Radiotherapy	Yes	9	25.7		6	17.1		0.56
	No	26	74.3		29	82.9		

### Intervention

In the intervention group, exergames using Nintendo Wii® were employed, and four games were selected (Wii Fit Aerobics—Basic Step, Wii Fit – Balance Games Penguin Slide, Wii Fit Super Hula Hoop, and Wi Fit Training Plus – Birds Eye Bulls Eye). In each session, the intervention group patients performed all four games. On the 2nd and 3rd postoperative days, the total intervention duration was 15 min; on the 4th and 5th postoperative days, it was 20 min; and on the 6th and 7th days, it was extended to 30 min. Patients were situated in front of a 42-in. LED television equipped with a Nintendo Wii® console. Prior to the commencement of the study, all participants received information about the games and how to operate the console. In the control group, standard care was maintained until discharge. In standard care, patients are encouraged to perform early mobilization and to walk down the corridor, continuing this practice until discharge.

### Instruments

The instrument was structured into two distinct parts: the first part related to socio-demographic and clinical characteristics, and the second part utilized the WHOQOL-BREF—Quality of Life Scale. The WHOQOL-BREF assesses the quality of life through 26 items. It is a Likert scale with five response options for each item, where the patient chooses the option that best suits them. The higher the score assigned to each item, the better the quality of life. This scale encompasses four domains: physical, psychological, social relationships, and environment. The scale has been validated for the Portuguese population [17]. Data collection by the researcher for both groups occurred at three different time points: upon admission, 48 h postoperatively, and on the 7th day after surgery. The game time and score obtained were also monitored (Table 4).

### Statistical analysis

The statistical analysis was conducted using the Statistical Package for the Social Sciences (SPSS), version 26. Group homogeneity tests were performed. A value less than 0.05 was considered to indicate statistical significance (*p*) for both parametric and non-parametric analyses. The chi-square test was used for the qualitative (categorical) variables. Repeated measures ANOVA was not used because the data did not meet normality criteria and there were only three observations, which is very few for repeated measures. In addition, we only wished to compare the two groups. Therefore, the Mann–Whitney test was used for the comparisons.

A linear regression was carried out, estimating for each regression model a binary variable relating to the group (control and intervention) as the explanatory variable and with the variables relating to the patients included as covariates to adjust for their effect on the value of the scale and thus be able to measure the effect of the intervention (Table 5).

### Ethics statement

The study participant was informed regarding the following: the identity of the study investigator, the study’s objectives, and other relevant information, such as the right to access and correct the information. Written informed consent was obtained from all study participants, and patient participation was voluntary. This study was performed in line with the principles of the Declaration of Helsinki. The study received approval from the Human Ethics Committee of the institution (171/2022-Ethics Committee of the Portuguese Institute of Oncology of Porto). Additionally, written approval was obtained from the institution’s board of directors.

## Results

### Participants’ characteristics

The presentation and analysis of results refer to 70 patients who underwent major abdominal surgery. This sample was divided into two groups: 35 patients in the control group and 35 patients in the intervention group.

Considering socio-demographic characteristics, in the control group the majority of individuals were male (25 people), with ages ranging from 39 to 81 years (mean = 62.3). In the intervention group, most patients were also male (20 people), with ages ranging from 30 to 78 years (mean = 63.1). There were no statistically significant differences between the groups (*p* = 0.823).

Regarding the length of hospital stay, in the control group, the minimum number of days was 5, and the maximum was 21, with an average of 8.4 days. In the intervention group, the minimum stay was 5 days, and the maximum was 15 days, with an average of 7.7 days. The result of the Mann–Whitney test is not significant (*p* = 0.621), indicating that the average number of hospitalization days is equal in both groups.

Quality of life assessment was conducted using the WHOQOL-BREF Scale, and the results are presented in Tables 2 and 3.

**Table 2:** WHOQOL-BREF Scale—Levels

Levels	Control		Intervention	
	*n*	%	*n*	%
Evaluation 1				
Needs improvement	3	8.6	0	0.0
Regular	18	51.4	20	57.1
Good	13	37.1	15	42.9
Very good	1	2.9	0	0.0
Evaluation 2				
Needs improvement	6	17.1	2	5.7
Regular	20	57.1	24	68.6
Good	9	25.7	9	25.7
Very good	0	0.0	0	0.0
Evaluation 3				
Needs improvement	5	14.3	0	0.0
Regular	21	60.0	20	57.1
Good	9	25.7	15	42.9
Very good	0	0.0	0	0.0
Total	35	100.0	35	100.0

**Table 3:** Analysis of differences between the control group and intervention group in quality of life

WHOQOL-BREF (quality of life)	1st assessment		2nd assessment		3rd assessment	
	C	I	C	I	C	I
**WHOQOL global**	77.4 (mean)	79.3 (mean)	72.7 (mean)	74.7 (mean)	73.9 (mean)	79.6 (mean)
	11.1^SD^	9.3^SD^	12.2^SD^	8.6^SD^	11^SD^	8.1^SD^
	*p* = *0.447*		*p* = *0.496*		*p* = *0.016*	
**Physical domain**	78.5 (mean)	82.5 (mean)	63.8 (mean)	69.6 (mean)	69.5 (mean)	77.1 (mean)
	13.4^SD^	9.6^SD^	12.5^SD^	14.2^SD^	11.4^SD^	12.0^SD^
	p = 0.233		p = 0.075		p = 0.005	
**Psychological domain**	80.3 (mean)	82.6 (mean)	75.6 (mean)	80.5 (mean)	78.8 (mean)	83.6 (mean)
	11.9^SD^	10.0^SD^	13.4^SD^	11.5^SD^	12.6^SD^	10.3^SD^
	P = 0.485		*p* = 0.962		*p* = *0.141*	
**Social Relationships domain**	82.5 (mean)	84.8 (mean)	79.2 (mean)	81.7 (mean)	80.2 (mean)	84.2 (mean)
	12.9^SD^	10.6^SD^	16.8^SD^	9.8^SD^	15.5^SD^	10.4^SD^
	*p* = *0.485*		*p* = *0.962*		*p* = *0.375*	
**Environmental domain**	78.6 (mean)	79.0 (mean)	73.0 (mean)	75.8 (mean)	74.2 (mean)	78.9 (mean)
	12.2^SD^	9.7^SD^	13.1^SD^	10.1^SD^	11.0^SD^	10.6^SD^
	p = 0.841		p = 0.508		p = 0.087	

“*C*” represents the control group, “*I*” represents the intervention group, and “*SD*” stands for standard deviation. The “*p*” values indicate the statistical significance of the differences between the groups in each domain of the WHOQOL-BREF questionnaire

In terms of levels, in evaluation 1, in the control group, the majority have a regular quality of life (18 individuals or 51.4%), followed by a good quality of life (13 individuals or 37.1%), needing improvement (3 individuals or 8.6%), and very good (1 individual or 2.9%). In terms of levels, in evaluation 1, in the intervention group, the majority have a regular quality of life (20 individuals or 57.1%), followed by a good quality of life (15 individuals or 42.9%), and there is no one needing improvement or having a very good quality of life.

Looking at evaluation 3, in terms of levels, in the control group, the majority have a regular quality of life (21 individuals or 60%), followed by a good quality of life (9 individuals or 25.7%), and needing improvement (5 individuals or 14.3%), with no one having a very good quality of life. Looking at evaluation 3, in terms of levels, in the intervention group, the majority have a regular quality of life (20 individuals or 57.1%), followed by a good quality of life (15 individuals or 42.9%), and there is no one needing improvement or having a very good quality of life.

In the 1st assessment, the mean quality of life in the control group and intervention group was 77.4 and 79.3, respectively. In the 2nd assessment, the mean quality of life in the control group was 72.7, while in the intervention group, it was 74.9. On the 7th day postoperative (3rd assessment), the mean quality of life in the control group was 73.9, while in the intervention group, the result was 79.6. From Table 2, it can be observed that the test result is significant (*p* = 0.016), indicating that the average quality of life in the control group is worse (Table 3).

Regarding the various subdomains of the scale, it can be observed that in the physical domain, the mean in the control group is worse (*p* = 0.005). Concerning the overall result of the quality of life, it is noted that the effectiveness results from its impact on the physical domain.

The duration and score of the four games are characterized in Table 4.

**Table 4:** Characterization of the duration and score of the games

Coefficients	Game1		Game 2		Game 3		Game 4	
	Duration (min)	Score	Duration (min)	Score	Duration (min)	Score	Duration (min)	Score
Minimum	3.6	131.1	2.1	79.8	1.6	354.4	0.0	0.0
Maximum	6.3	651.4	4.3	218.7	4.6	3582.6	3.4	663.7
Average	5.6	360.9	4.0	146.0	3.7	2135.4	2.2	275.4
Standard deviation	0.65	120.1	0.47	30.4	0.87	703.2	1.1	201.8

In the physical domain, the adjusted model in the three evaluations showed that the group is significant, concluding that the average quality of life is worse in the control group in all three evaluations. In the psychological domain, the adjusted model in the three evaluations also showed that the group is significant, concluding that the average quality of life is worse in the control group in all three evaluations. In the social relations domain, the model adjusted for the three assessments showed that the group was not significant, so it is assumed that the average level of quality of life is the same in both groups in all three assessments. In the area of the environment, the model adjusted for the three assessments showed that the group is not significant, so it is assumed that the average level of quality of life is the same for both groups in all assessments. In the global field, the model adjusted in the second assessment showed that the group is not significant, so it is assumed that the average level of quality of life was the same for both groups in this assessment (Table 5). On the other hand, the group was significant for assessments 1 and 3, and it is concluded that the average quality of life is worse in the control group in these assessments.

**Table 5:** Linear regression analyses

WHOQOL-BREF						
Variable	Evaluation 1		Evaluation 2		Evaluation 3	
	Estimate	*p*	Estimate	*p*	Estimate	*p*
Physical domain						
Intervention group	9.452	0.002	8.333	0.015	7.781	0.022
Age	− 0.276	0.149	0.047	0.828	0.184	0.393
Gender	− 6.352	0.046	− 5.883	0.101	− 1.846	0.602
Marital status	− 1.147	0.782	2.178	0.643	− 1.735	0.711
Hospitalization length (days)	0.202	0.653	− 1.353	0.010	− 0.766	0.136
BMI	0.798	0.088	0.210	0.688	0.423	0.420
Metastatic disease	11.371	0.012	− 3.155	0.526	0.991	0.841
Chemotherapy neoadjuvant	− 6.177	0.155	3.766	0.441	− 0.324	0.947
Chemotherapy	3.718	0.360	8.483	0.068	5.555	0.228
Psychological domain						
Intervention group	6.559	0.018	8.227	0.016	7.536	0.022
Age	− 0.139	0.426	− 0.092	0.669	− 0.106	0.610
Gender	− 9.525	0.002	− 7.519	0.037	− 3.942	0.253
Marital status	2.788	0.465	3.529	0.453	3.283	0.471
Hospitalization length (days)	0.035	0.932	− 0.779	0.130	− 0.245	0.620
BMI	1.133	0.010	0.506	0.335	0.864	0.092
Metastatic disease	6.490	0.111	4.724	0.343	6.871	0.157
Chemotherapy neoadjuvant	0.129	0.974	− 3.503	0.473	0.589	0.901
Chemotherapy	0.372	0.920	1.937	0.673	− 0.010	0.998
Social relationships domain						
Intervention group	4.507	0.168	3.505	0.387	5.009	0.183
Age	0.023	0.915	− 0.116	0.656	0.016	0.948
Gender	1.369	0.692	− 2.572	0.550	5.575	0.164
Marital status	1.674	0.715	− 6.089	0.287	− 2.032	0.700
Hospitalization length (days)	0.138	0.781	− 0.372	0.548	0.159	0.781
BMI	0.598	0.244	0.694	0.277	0.608	0.302
Metastatic disease	− 3.142	0.435	0.330	0.947	− 0.898	0.846
Chemotherapy neoadjuvant	1.533	0.751	2.537	0.673	4.997	0.371
Chemotherapy	2.425	0.610	− 0.613	0.917	− 0.341	0.950
Intervention group	− 1.837	0.681	5.832	0.297	4.804	0.352
Environmental domain						
Intervention group	4.566	0.104	4.383	0.193	5.838	0.061
Age	− 0.115	0.522	− 0.142	0.513	− 0.030	0.878
Gender	− 1.693	0.567	− 1.867	0.600	− 0.332	0.919
Marital status	3.918	0.318	3.555	0.452	3.710	0.393
Hospitalization length (days)	− 0.122	0.773	− 0.743	0.150	− 0.346	0.462
BMI	0.858	0.053	0.230	0.662	0.699	0.151
Metastatic disease	0.049	0.989	2.060	0.619	− 0.500	0.895
Chemotherapy neoadjuvant	6.175	0.139	− 0.027	0.996	5.625	0.222
Chemotherapy	− 1.070	0.792	2.771	0.572	1.289	0.774
Intervention group	− 3.173	0.407	− 2.455	0.595	− 5.013	0.239
Total scale						
Intervention group	5.450	0.030	3.975	0.184	7.269	0.010
Age	− 0.094	0.555	− 0.138	0.472	− 0.044	0.807
Gender	− 5.226	0.050	− 3.450	0.277	0.382	0.896
Marital status	0.308	0.929	3.086	0.462	3.466	0.374
Hospitalization length (days)	− 0.284	0.451	− 0.773	0.093	− 0.558	0.189
BMI	1.083	0.007	0.413	0.378	0.552	0.206
Metastatic disease	3.994	0.279	1.363	0.758	4.296	0.298
Chemotherapy neoadjuvant	0.387	0.915	0.325	0.940	1.190	0.768
Chemotherapy	0.142	0.967	2.009	0.623	0.707	0.852

## Discussion

Exergames, a fusion of digital gaming and physical activity, have gained significant traction as a rehabilitation tool in the healthcare sector. This research focused on evaluating the impact of exergame-based rehabilitation on the life quality of cancer patients who have had abdominal surgery. The use of technology in rehabilitation is progressively growing, and emerging technologies, particularly exergames, encourage patients to engage even more in their daily treatment [14, 18]. Exergaming combines exercise with gaming, where patients utilize physical movements to interact with the game, and it has been utilized in cancer patients [9, 14]. Despite the increasing popularity of these resources, no published reports discuss the efficacy of exergames on the quality of life of patients after abdominal oncologic surgery.

In the specific case of cancer patients, they present symptoms related to the disease, such as decreased functionality, fatigue, reduced strength, pain, insomnia, and mood disturbances, with significant implications for their quality of life and overall well-being [7]. Studies suggest that rehabilitation programs in cancer postoperative care lead to clinically relevant improvements, particularly in physical functions and various dimensions of quality of life, as well as in overall health status after rehabilitation or shortly after the treatment [18, 19]. Rehabilitation is crucial after cancer surgery, and the use of exergames can be an important resource for postoperative recovery [14].

Research indicates that exergames positively affect motivation for active participation in rehabilitation and impaired functions [6, 9]. Exergames have demonstrated several advantages in rehabilitation, including lowering the pain threshold and providing psychological and social interaction benefits for patients [11, 12, 14]. These advantages stem from the unique features of exergames that combine physical activity with gaming elements, making them motivating for patients [14]. They also offer other benefits, such as the ability to customize difficulty levels, provide feedback, and monitor activity [20].

Our results suggest that the use of an exergame may contribute to an improvement in average quality of life in the intervention group, compared to standard care. Quality of life measures have become a vital and often required part of health outcomes appraisal [21–23].

This impact is mainly due to its effect on the physical domain. As these authors have pointed out, exergaming proves to be a promising tool for improving the physical health of cancer patients [6, 7, 12, 21, 24, 25]. Although conducted in a different population, these authors have demonstrated that exergames, when used as a rehabilitation method, can offer an interactive alternative intervention with positive outcomes for quality of life [22]. The demand for cancer rehabilitation following surgery is projected to rise significantly due to the aging population and an increasing number of cancer survivors. These patients experience a wide range of physical limitations and symptoms that detrimentally impact their health and quality of life [22, 26]. The results indicate that the use of exergames is beneficial, improving QoL in these patients, which is in line with studies that analyze quality of life and exercise in cancer patients [25, 27]. One possible explanation is that exercise helps alleviate the stress of cancer symptoms, a critical factor in QoL [27]. Exercise releases dopamine, improving mood and managing negative emotions, as well as strengthening the immune system, improving overall physical fitness [27].

Despite the numerous advantages of immediate postoperative rehabilitation, which directly influences quality of life, some authors have reported low adherence rates among this patient group [26]. A primary challenge in physical rehabilitation treatments lies in maintaining patient motivation, as these treatments involve slow, repetitive, and often uncomfortable movements [28, 29, 30]. Exergames have shown promising results in enhancing patient motivation to engage in rehabilitation therapies [14, 28]. It is essential to note that while exergames can be beneficial in abdominal surgery rehabilitation, they should be supervised and prescribed by healthcare professionals to complement other methods. Individual patient needs and conditions should always be considered when integrating exergames into the rehabilitation program.

Although the results of this study demonstrate the potential applicability of exergames in an emerging area with limited evidence of their use, such as major abdominal surgery rehabilitation, further studies, including more clinical trials, should be conducted. It would also be important to determine the best way to incorporate exergames into rehabilitation programs, the ideal protocol for duration, and the frequency of execution for the patient [6].

Despite the strengths of the study, some limitations should be considered for the generalization of the results. First, the limited number of participants. Additionally, there was variability in some of the pathologies, mainly intestinal or gastric. Moreover, the fact that the Nintendo Wii® platform was used and not an exergame specifically designed for this purpose. However, it is important to recognize that another limitation of this study is the lack of previous research in this area that specifically looks at the use of exergames in people with cancer. And those that exist are aimed at the rehabilitation of people with breast cancer [14, 25]. Although some authors reference in review studies that it has not yet been possible to reach a conclusion on the efficacy of a single exergame or specific console, but rather exergaming as a whole [7, 14].

## Key findings

- The rehabilitation of cancer patients has specific characteristics that must be considered by health professionals.
- Exergames should continue to be integrated as an appealing method for post-abdominal surgery rehabilitation.
- High-quality clinical trials, including larger sample groups with reduced risk of bias, should be conducted.

## Conclusion

The results of this investigation suggest that the use of an exergame, such as Nintendo® Wii, is a valuable tool for the rehabilitation of cancer patients who have undergone abdominal surgery, bringing potential benefits to their quality of life, especially in the physical domain. Further studies are needed to validate the most suitable protocol for this population, including the types of games used, the duration, and frequency of the intervention.

However, the potential of exergames can help motivate and challenge individuals in the rehabilitation process following abdominal oncologic surgery. This will be an important challenge to be collectively addressed, considering new intervention resources, including remote approaches, for a paradigm shift toward hybrid care.

## Data Availability

No datasets were generated or analysed during the current study.
